# Clean birth kits to improve birth practices: development and testing of a country level decision support tool

**DOI:** 10.1186/1471-2393-12-158

**Published:** 2012-12-19

**Authors:** Vanora A Hundley, Bilal I Avan, Haris Ahmed, Wendy J Graham

**Affiliations:** 1 School of Health & Social Care, Bournemouth University, Royal London House, Christchurch Road, Bournemouth, BH1 3LT, UK; 2 NMAHP Research Unit, University of Stirling, Stirling, FK9 4LA, UK; 3 Department of Disease Control, London School of Hygiene & Tropical Medicine, Keppel Street, London, WC1E 7HT, UK; 4 Pathfinder International, Islamabad, Pakistan; 5 Immpact, University of Aberdeen, Health Sciences Building, Foresterhill, Aberdeen, AB25 2ZD, UK

**Keywords:** Clean birth practices, Birth kits, Decision support tool

## Abstract

**Background:**

Clean birth practices can prevent sepsis, one of the leading causes of both maternal and newborn mortality. Evidence suggests that clean birth kits (CBKs), as part of package that includes education, are associated with a reduction in newborn mortality, omphalitis, and puerperal sepsis. However, questions remain about how best to approach the introduction of CBKs in country. We set out to develop a practical decision support tool for programme managers of public health systems who are considering the potential role of CBKs in their strategy for care at birth.

**Methods:**

Development and testing of the decision support tool was a three-stage process involving an international expert group and country level testing. Stage 1, the development of the tool was undertaken by the Birth Kit Working Group and involved a review of the evidence, a consensus meeting, drafting of the proposed tool and expert review. In Stage 2 the tool was tested with users through interviews (9) and a focus group, with federal and provincial level decision makers in Pakistan. In Stage 3 the findings from the country level testing were reviewed by the expert group.

**Results:**

The decision support tool comprised three separate algorithms to guide the policy maker or programme manager through the specific steps required in making the country level decision about whether to use CBKs. The algorithms were supported by a series of questions (that could be administered by interview, focus group or questionnaire) to help the decision maker identify the information needed. The country level testing revealed that the decision support tool was easy to follow and helpful in making decisions about the potential role of CBKs. Minor modifications were made and the final algorithms are presented.

**Conclusion:**

Testing of the tool with users in Pakistan suggests that the tool facilitates discussion and aids decision making. However, testing in other countries is needed to determine whether these results can be replicated and to identify how the tool can be adapted to meet country specific needs.

## Background

Sepsis is one of the leading causes of both maternal [1] and newborn mortality [2,3]. Ensuring that birth practices are clean is estimated to reduce neonatal mortality due to tetanus by 30% in home births and 38% in facility births [4]. Clean birth practices, combined with monitoring and active third stage management, may prevent up to 23% of maternal deaths in low income countries [5]. Achieving a clean birth requires the application of skills by the care provider and access to essential supplies, such as soap. For several decades clean birth kits (CBKs) have been recommended as a means of ensuring those supplies. The World Health Organization (WHO) has supported CBK use as a means by which to explicitly “strengthen standards of cleanliness” in home deliveries [6] and within health facilities that lack the capacity to sterilise equipment [7]. A recent systematic review found that CBKs, as part of package that included education, were associated with reduced newborn mortality, omphalitis, and puerperal sepsis [8]. It also highlighted the heterogeneity in terms of the contents of CBKs and the methods by which the CBK was distributed; raising questions about how best to approach the introduction of CBKs in country. We set out to develop a practical tool, based on the available data, for use at national and sub-national level. The tool was aimed at policy makers and programme managers; specifically those decision makers planning the overall strategy for care at birth, which would include encouraging women to give birth in facilities and improving supply and demand of commodities at the lowest level of community and facility health care.

Pakistan was chosen for the test of the decision support tool as it is representative of a number of countries making progress in maternal mortality reduction, but unlikely to meet the Millennium Development Goal (MDG) target set for 2015. Pakistan has an estimated maternal mortality of 260 per 100,000 live births [9]; an estimate that reflects a 48% reduction in maternal mortality since 1990 [9], but indicates that accelerated progress is needed to achieve the target set for 2015. The maternal mortality ratio (MMR) is significantly higher in rural areas, largely as a consequence of inequities in health service utilisation [10]; recent figures show that only 30% of births in rural areas were attended by a skilled care provider compared with 60% in urban areas [11]. In Pakistan, sepsis is the second leading cause of both maternal (13.7%) and neonatal mortality (20%) [10]. A recent survey suggests that failures in hygienic practices may contribute significantly to the problem [12]. Hassan et al. found that unhygienic practices were common among traditional birth attendants (TBAs), but were also evident among skilled birth attendants working in Sindh province [12]. A lack of resources and educational requirements has also been implicated in the high neonatal mortality rates [13,14]. CBKs have been recommended as a method of overcoming the challenges of accessing essential supplies in Pakistan, and along with education, could be a valuable tool in promoting safe childbirth [12].

**Figure 1:**
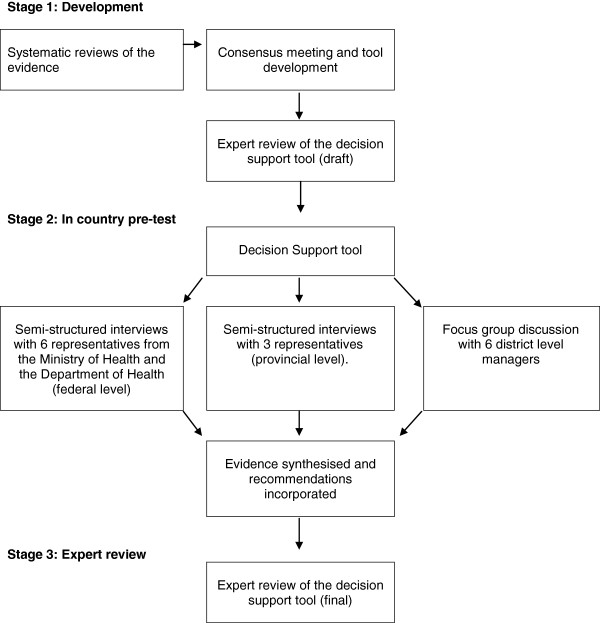
Development and testing of the decision support tool.

This paper reports the development and testing of the decision support tool; a three-stage process involving an international expert group and country level testing (Figure 1). The aim was to produce a practical tool for use by policy makers and programme managers who are considering the potential role of CBKs in their strategy for care at birth.

## Methods

### Tool development

Development of the decision support tool was undertaken by the Birth Kit Working Group (BKWG) (Table 1) and involved a review of the evidence, a consensus meeting, drafting of the proposed tool and expert review. Members of the BKWG undertook two substantial literature reviews to examine the issues surrounding clean birth practices and the effectiveness of CBKs [4,8]. The international, multidisciplinary BKWG (comprising clinicians, researchers, policy makers and programme managers) met on two occasions to review the evidence, discuss the issues and draft an outline for the decision support tool. Tool development was conducted by a smaller working group, and the tool was then circulated to experts for peer review (Table 1) and amendments made.

**Table 1:** Group members

*Members of the Birth Kit Working Group:*	Priya Agrawal (Harvard School of Public Health), Haris Ahmed (Pathfinder), Bilal Avan (IDEAS, LSHTM), Ann Blanc (MHTF), Hannah Blencowe (LSHTM), Thomas Burke (Harvard Medical School), Oona Campbell (LSHTM), Mickey Chopra (UNICEF), Patricia Coffey (PATH), Anthony Costello (ICH/UCL), Simon Cousens (LSHTM), Jo Cox (MM Campaign), Susan Crane (ICH/UCL), Luc de Bernis (UNFPA), Luis Andres de Francisco Serpa (WHO), France Donnay (Gates Foundation), Mark Dybul (Georgetown University), Melody Eckardt (Harvard Medical School), Helga Fogstad (Norad), Lynn Freedman (AMDD), Wendy Graham (Immpact), Homaira Hanif (JHU), Elizabeth Hoff (Millenium Foundation), Vanora Hundley (Bournemouth University), Jessica Hulse (SNL), Lily Kak (USAID), Asma Khalid (MSI), Louise Kleberg (Millenium Foundation), Joy Lawn (SNL), Helena Lindborg (DFID), Elizabeth Leahy Madsen (MHTF), Nahed Matta (USAID), Zoe Matthews (University of Southampton); Peter McDermott (CIFF), Claudia Morrissey (SNL), Luke Mullany (JHU), Anne Pfitzer (SNL), Melanie Ridge (ICH/UCL), Craig Rubens (GAPPS), Lale Say (WHO), Unni Silkoset (Norad), Ann Starrs (FCI), Catherine Taylor (PATH), Steve Wall (SNL), Eva Weissman (Futures Institute), and Peter Winch (JHU).
*Members of the tool development group:*	Haris Ahmed (Pathfinder), Bilal Avan (IDEAS, LSHTM), Hannah Blencowe (LSHTM), Wendy Graham (Immpact), Vanora Hundley (Bournemouth University), and Joy Lawn (SNL).
*Expert reviewers in stage 1:*	Ann Blanc (MHTF), Melody Eckardt (Harvard Medical School), Asma Khalid (MSI), Claudia Morrissey (SNL), and Steve Wall (SNL).

The tool comprised three separate algorithms to guide the policy maker or programme manager through the specific steps required in making the country level decision about whether to use CBKs. In order to help the user identify the required information, each algorithm was followed by a series of questions that could be administered by interview, focus group or questionnaire.

**Figure 2:**
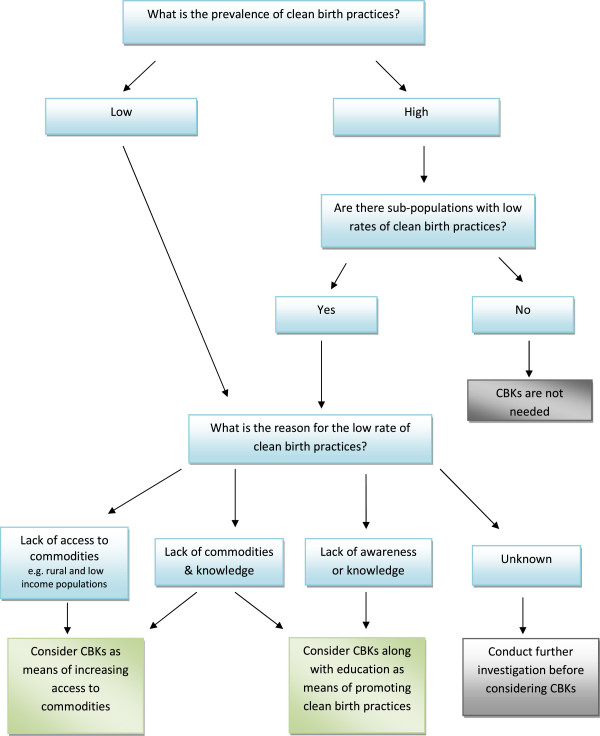
Algorithm 1 – establishing whether there is a need for clean birth kits.

Algorithm 1 - *establishing whether there is a need for clean birth kits* (Figure 2) - Evidence from the two systematic reviews [4,8] demonstrated a significant role for CBKs in promoting clean birth practices, thus algorithm 1 focuses on the prevalence of clean birth practices and whether there is a need to consider using CBKs. This includes identifying whether there are sub-populations with low rates of clean birth practices.

**Figure 3:**
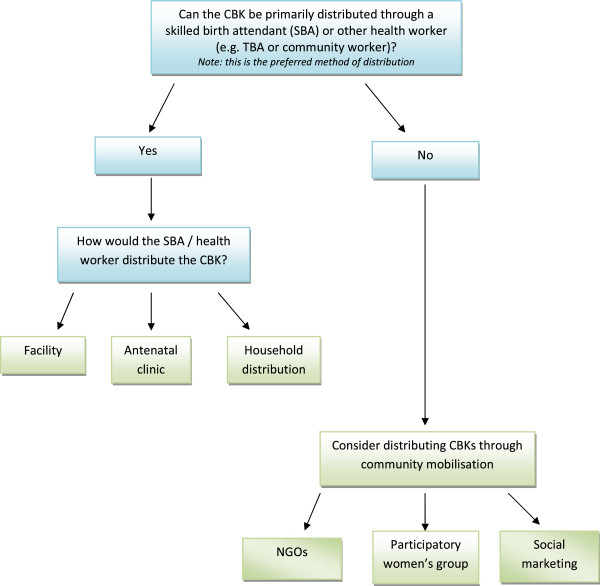
Algorithm 2 – identifying how clean birth kits will be distributed.

Algorithm 2 - *identifying how clean birth kits will be distributed* (Figure 3) - Having identified a role for CBKs in improving clean birth practices, the decision maker needs to decide how best to distribute CBKs in country. Algorithm 2 guides the decision maker through the possible routes of distribution that were identified from the review of literature [15]. The aim is to identify a route that is appropriate to the country specific context (e.g. taking into account considerations such as geography and infrastructure).

**Figure 4:**
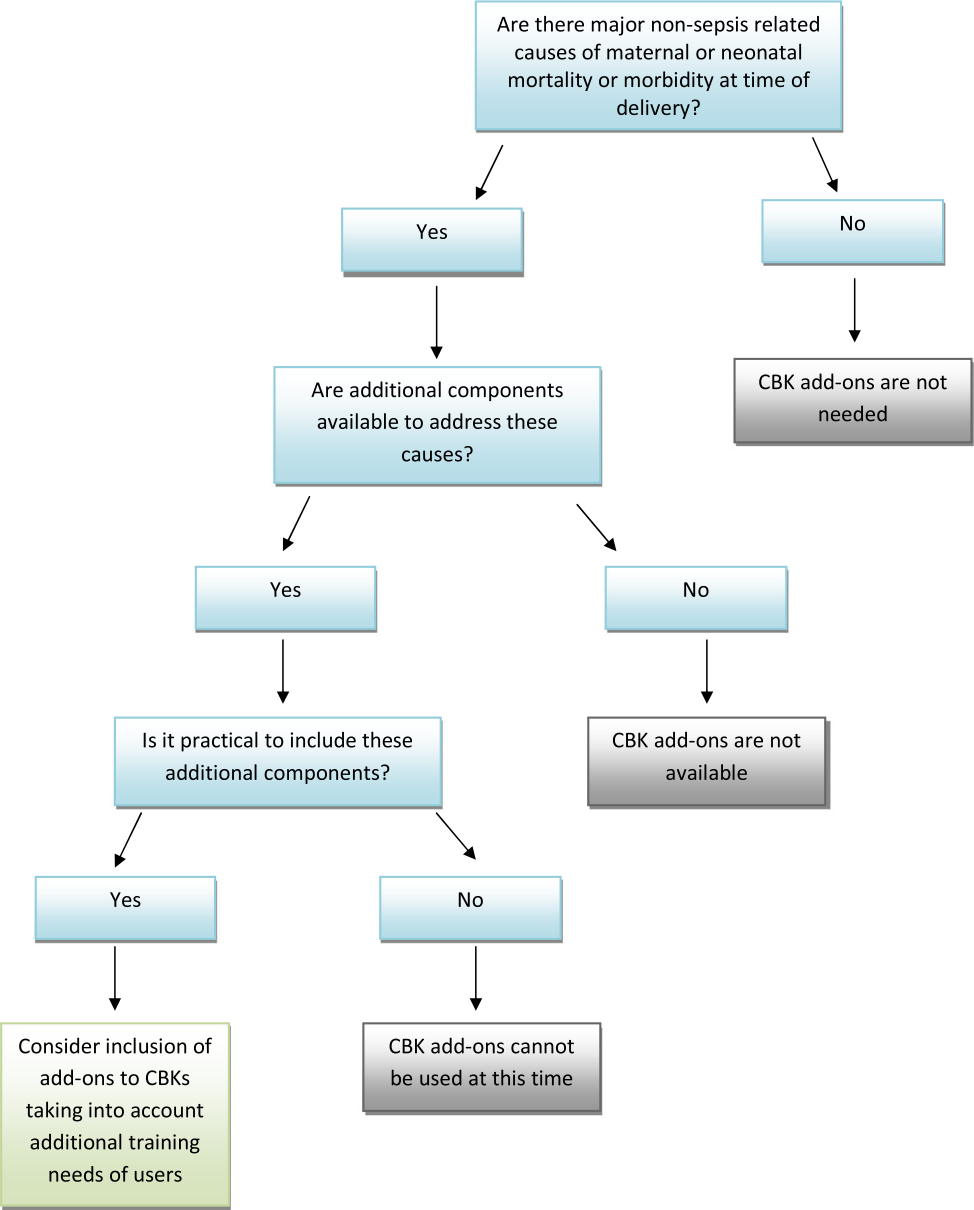
Algorithm 3 – identifying whether there is a need for additional components.

Algorithm 3 - *identifying whether there is a need for additional components* (Figure 4) – Here the decision maker is asked to look at whether non-sepsis related causes of mortality and morbidity could be addressed by adding relevant components to the CBK. An example of such a component is misoprostol for the prevention of postpartum haemorrhage. The intention is for the decision maker to identify components that address country specific needs.

If the decision is supportive of a potential role for CBKs, a further step would be required to explore practical considerations such as procurement, cost and issues relating to utilisation (e.g. training). This would also include the potential for evaluation of the programme. Details of this step are not included in the tool, as they would need to be country specific and take into account existing protocols regarding the health system and supply chains. The final versions of the algorithms appear in Figures 2,3,4. The full decision support tool is available on the Immpact web site [16].

### Country level test of the tool

Qualitative methods were used to conduct the test with policy makers and programme managers in Pakistan [17,18]. Purposive sampling ensured that both federal and provincial level decision makers were included in the study [17]. Semi structured interviews were conducted with six representatives from the Federal Ministry of Health in Islamabad and with three representatives from the Provincial Department of Health Punjab in Lahore. The opinion of district level management was sought through a focus group discussion (FGD) conducted in the Chakwal district (six participants). The process involved participants working through the decision tool in the presence of the interviewer and reflecting on the ease of completion and the value of the tool. Ethical approval was sought but, because the interviews involved health service officials and not patients, we were advised instead to seek formal permissions from the health departments involved and these were obtained. All participants gave informed consent prior to the interviews.

The interview schedule was drafted by the field coordinator (HA) under the guidance of the BKWG members and followed the format of the decision support tool. The schedule allowed the participants to work through the decision support tool in the presence of the interviewer, and to identify areas that needed clarification. The interview schedule was piloted [19] with two participants in private practice.

Focus group interviews followed a similar format, with the algorithms being presented to the participants to stimulate discussion. However, the discussion was more open with the facilitator using the questions within the decision support tool as prompts if required.

Two interviewers were identified by the field coordinator based on their local knowledge and experience in conducting interviews and focus group discussions. The interviewers worked together as a team, and this enabled the interviews to be recorded by hand since participants preferred not to be tape recorded. All participants were informed about the nature and purpose of the study prior to the interview. They were assured that their responses would remain confidential, and that they would not be identifiable in study reports. The interviews were recorded verbatim in Urdu and English, and then translated into English for analysis. Responses were categorised according to the algorithms to which they related and the specific steps within those algorithms [20]. Quotes were selected to illustrate the themes from participants’ responses. For the purposes of reporting, interview quotes are labelled as P1-P9 for interview participants and FG1-FG6 for focus group participants.

The process of developing the decision support tool, testing and amending it was detailed as part of the minutes of the BKWG and served as an audit trail for the work.

## Results

### Participants

All nine interviewees held positions as policy makers or programme managers within the Department or Ministry of Health (six in the Federal Ministry of Health in Islamabad and three in the Provincial Department of Health Punjab in Lahore). Four had been in post for more than two years, three for one year and two for less than a year. Seven of the participants were male and two were female. It is not possible to give details of their position in order to maintain confidentiality. The six focus group participants all held positions within district level management in the Chakwal district. Five were male and one was female. All had responsibility for public health.

### Tool as a whole

The country level testing revealed that the decision support tool was easy to follow and helpful in making decisions about the potential role of CBKs in their country’s strategy for care at birth.

"“very helpful, self-explanatory and thought provoking.” (P5)"

"“very helpful. It gives you food for thought.” (P2)."

Some participants reported that the tool was particularly helpful in initiating discussion: “*It made the concept much clearer. The thoughts are there – clarity to bring it on track*.” (P4)

### Algorithm 1

The main challenge for participants in using algorithm 1 was in identifying the relevant data about the prevalence of clean birth practices and CBK use. All participants reported that the proportion of clean births in Pakistan was very low. When asked to provide a figure, six of the interview participants estimated that 30-40% of women had access to clean birth practices. The FGD participants also came to a consensus on 30-40%, while the remaining three interview participants suggested lower estimates of between 10-15%. One participant noted that clean birth practices were lower in rural areas and in home deliveries in particular:

"“…in home delivery only 10% deliveries will have these cleans [the six cleans] 90% will not observe the practices.” (P5)"

Focus group participants reported that although there was a problem with births attended by unskilled attendants, skilled attendants also had lapses in clean birth practices:

"“Even our Doctors don’t take care of hand washing. It’s a very simple and economical method but it is not taken care of.” (FG1)"

Feedback on algorithm 1 confirmed a role for CBKs beyond just providing commodities. Participants agreed that CBKs could have an important role in raising awareness among women, as well as birth attendants, of the need for clean birth practices:

"“It’s a chain of education ….When you are giving something then you are also going to tell them what to do with it and how to use it.” (P1)"

However, participants had mixed views with regard to whether CBKs could act as an incentive to facility birth. One participant cited an example where a CBK, introduced by a local non-government organisation (NGO), acted as an incentive for women to go to a facility. Others were less certain. One stated:

"“I don’t think so. …. If the kit is there then at least the kit will be utilized wherever she goes for delivery. But the presence of kit only cannot be the incentive. Bigger motivation is SBA [Skilled Birth Attendant].” (P5)"

Most participants thought that CBKs would not act as a disincentive for facility birth. However, one participant expressed caution and suggested that the method of distribution would need to be tested:

"“It can happen they [women] may say why go to anyone if dai^*a*^has this, let’s have delivery from her. If this is looked at through a short study it will be better.” (P1)"

FGD participants felt that the distinction between ‘lack of commodities’ and ‘lack of awareness’ could be clearer in algorithm 1 and recommended a minor modification so that both an individual option and a joint option were available.

### Algorithm 2

The issue of distribution, explored using algorithm 2, proved challenging for participants. The policy makers at the federal level expressed less certainty about the distribution and use of CBKs than policy makers and implementers at provincial level or FGD participants.

Skilled birth attendants (SBAs) were identified to be the main users or recipients of the CBKs. Pregnant mothers were identified as users by some participants and a few considered Dais or TBAs. FGD participants noted that although there was a move to replace TBAs with SBAs, if CBKs were made readily available to TBAs then infection rates would be greatly reduced. Both interview participants and FGD participants believed that training or refresher training would be required for the users of the CBKs.

Most of the participants were in favour of distributing the CBK at household or community level. Various suggestions included giving CBKs direct to women at registration, via antenatal clinics, health facilities, shops and pharmacies, and through private doctors. However, some participants noted that the approach should not be limited to one method:

"“It should be a mixture where the facility is there or not there, availability should be universal.” (P1)"

"“You cannot categorize any one [approach] it has to be a multi-pronged approach.” (P4)"

Although identifying who should distribute CBKs was not easy, most participants felt that Lady Health Workers (LHWs) would be the most appropriate group, since they interfaced directly with the population at community level. LHWs are female community health workers who receive training, medical supplies and a small allowance from a government health facility to which they are attached [21]. LHWs provide essential primary health services such as health education, antenatal and postnatal care, and family planning services. Both interview participants and focus group participants felt that this was the most appropriate method of CBK distribution because LHWs covered a large amount of the population. Comments included: 

"“their [LHWs] responsibility is to be present at the time of birth.” (P1)"

"“Structure wise LHWs are strong and can be done substantially through them.” (P4)"

"“There will be extra cost involved if it is other than LHWs.”(P5)"

There were no problems with the flow or wording of algorithm 2. Participants found the tool useful in helping to think about the best way to get CBKs to potential users.

### Algorithm 3

Discussion around algorithm 3 focused on the wish list versus the realistic list:

"“the things related to common causes should be added, otherwise it can be suggested to put a gynecologist also in the kit, if possible!” (P9)."

Other concerns related to the importance of planning a programme of training to accompany any additions to the kit. For example, a number of participants mentioned that misoprostol might be included in a CBK if birth attendants received a programme of training first. However, participants felt that the algorithm did draw the decision maker’s attention to issues of practicality and training.

Participants recognised that the decision support tool could not be used in isolation and that other components, such as training, would be important in helping policy makers reach decisions about strategies for care at birth. Some participants suggested that the data needed for the algorithms (such as mortality rates, number of skilled birth attendants and costs) would need to be collected in advance of the discussions: “*This will need a detailed review*.”(P1). It was suggested that country specific data sources could be included in an appendix to the tool.

The findings from the country level testing were presented and reviewed at a meeting of the BKWG. Minor modifications were agreed to clarify algorithm 1 and the guidance for algorithm 3, however the group decided that country specific data sources could not be linked to the tool since each country was likely to require a different set of data. Instead it was agreed that a generic list of resources would be added as an appendix to the tool.

## Discussion

The test indicated that policy makers and programme managers found the decision support tool useful in stimulating discussion around the role of CBKs for country level strategies for care at birth. Previous studies have shown that CBKs (a collection of disposable commodities) are effective at promoting clean birth when used as part of a strategy that includes education [8]. The tool helped policy makers to identify whether the prevalence of clean birth practices was low and if so to identify the reason for this (access to commodities, awareness and/or knowledge). Using the second algorithm participants were able to conceptualise how CBKs could be used to as a means of distributing commodities in areas where supply chains are erratic. There is evidence that the performance of health systems in low income countries may be marred by several factors including lack of adequate resources, infrastructure, coordination and management [22]. However, a lack of supplies and commodities is central to the ineffective utilisation of health services and deters clients, mainly women and children, from making contact with public health facilities or staff. Consequently those who can afford it resort to private health systems to provide the necessary medical commodities, but such services are unregulated, over-priced [23] and can pose serious health challenges [24].

Although extra resources are required to meet medical commodity needs, effective supply chain management is a necessary prerequisite and requires strengthening and innovative approaches to address the gap [25]. CBKs are one potential strategy to facilitate the packaging of medical supplies together thereby ensuring a clean, and hence safe, delivery process. Such commodities might be included within the CBK, as in the recent study of chlorhexadine [26], or be ‘bundled’ with a CBK for distribution. In developing the decision support tool we acknowledged the fact that the distribution mechanism and content should be congruent with regional variations in community needs and health systems. Hence, the tool is especially useful for decentralised health systems where some key decision making about the nature and content of the health service takes place locally.

It is important to highlight that a decision support tool, aimed at increasing access to commodities, cannot on its own be sufficiently effective in making positive changes in maternal and newborn outcomes in low income countries [27,28]. Such tools need to be used alongside a regular supply chain, community mobilisation and health staff training. Participants in our test reported that the decision support tool enabled them to discuss how CBKs could be used to raise awareness of clean birth practices through existing health systems, and how a CBK could be modified to incorporate educational interventions to facilitate these practices. However, they recognised that the tool was only one component in the strategy to improve care at birth.

Although the test was successful, the policy makers and programme managers recognised the need for country level data to support these discussions. Participants acknowledged that they often did not have data to support their estimates. We did not attempt to verify the estimates given or to measure the prevalence of clean birth practices in country. Instead our aim was to guide the policy maker or programme manager through a series of steps, which could require the use of existing data, in order to make decisions about whether to use CBKs. Further research is needed to explore how national level evidence synthesis can be incorporated into the process and how the tool can be adapted to meet district needs.

## Conclusion

We have developed a decision support tool for policy makers and programme managers who are considering the potential role of CBKs in their strategy for care at birth. Testing of the tool in Pakistan suggests that the tool facilitates discussion and aids decision making. However, further testing in other countries is needed to determine whether these results can be replicated and to identify how the tool can be adapted to meet country specific needs.

## Endnotes

^a^An untrained traditional birth attendant.

## Competing interests

The authors declare that they have no competing interests.

## Acknowledgements

This decision support tool was produced thanks to the experience, ideas and input of many different people and organisations. The Birth Kit Working Group was instrumental to its development, testing and review – the members are listed in Table 1. We are also grateful to all the decision makers who gave their time to test the decision support tool in country. Thanks are also due to our two interviewers Dr Munazza Haris and Dr Nadeem Hassan. This study was funded by the Maternal Health TaskForce, EngenderHealth. The funding body had no role in the design or conduct of the study, or in the writing of the paper. Funding for the article-processing charge is through the primary author’s institution, Bournemouth University.

## Authors’ contributions

All authors were involved in the design of the decision support tool and the plan for the test. VH co-ordinated the expert group review. The interview schedule for the country test was drafted by HA with support VH and BA. HA and VH supervised the recruitment and interview process, and undertook the preliminary analysis. All authors were involved in the synthesis of data and finalising the tool. VH produced the first draft of the paper. All authors contributed to the writing and approved the final version.

## Pre-publication history

The pre-publication history for this paper can be accessed here:

http://www.biomedcentral.com/1471-2393/12/158/prepub
